# Novel Bedside Utilization of Foley Catheter in the Emergent Removal of Colorectal Foreign Body: A Case Report and Literature Review

**DOI:** 10.7759/cureus.20217

**Published:** 2021-12-06

**Authors:** Terry Lefcourt, Andrew Ku, Leo Issagholian, Arianna S Neeki, Milton Retamozo, Fanglong Dong, Michael M Neeki

**Affiliations:** 1 Emergency Medicine, Arrowhead Regional Medical Center, Colton, USA; 2 Neurosurgery, California University of Science and Medicine, Colton, USA; 3 Emergency Medicine, California University of Science and Medicine, Colton, USA; 4 General Surgery, Arrowhead Regional Medical Center, Colton, USA; 5 General Surgery, California University of Science and Medicine, Colton, USA

**Keywords:** emergency department, foley catheter, object in anus, rectal foreign body, rectal foreign object

## Abstract

Rectal foreign bodies (RFBs) present unique challenges to the emergency physician. Failure to emergently remove the RFB may lead to additional intraoperative procedures with increased likelihood of complications. We present a case of retained RFB in the emergency department, in which the usual standard approaches to transanal removal had failed. A last-ditch effort by utilizing a Foley catheter inside the object rather than around it led to the successful removal of the RFB. An intense review of the literature highlights the importance of using various novel applications of a Foley catheter to consider cases of RFBs.

## Introduction

Rectal foreign bodies (RFBs) are defined as objects that were either inserted through the rectum or ingested and become lodged in the distal descending colon [1,2]. Records of RFB presentations date back to the 16th century, making them a historic medical complaint which future physicians will likely encounter [3]. The incidence of RFBs needing medical intervention is estimated at 0.15 per 100,000 persons annually [4]. Males aged 30-50 years are at an overwhelmingly higher rate of association with RFBs than females [5,6]. Lake et al. reported 93 RFB encounters across two hospitals between 1993 and 2002 [7]. RFBs may be localized during a digital examination, using various imaging modalities, or even incidentally when administering enemas [2,6]. Difficulty in early diagnosis was noted in prior publications when patients were withholding critical information that would reveal the presence of RFBs [8]. In general, the most common presenting complaint may be nonspecific abdominal pain with constipation [9].

Insertion of RFBs can be due to several reasons, including sexual gratification, drug concealment, or sexual assault [2,6,10]. The majority of RFBs are commercially manufactured by known manufacturers as sexual devices and glass objects but they can also include fruits, vegetables, containers, other articles, and homemade objects [5,9-12]. In addition, the wide varieties of RFBs in patient presentations can make standardizing removal techniques difficult due to varying shapes, sizes, depth of insertion, or other intrinsic factors [4,6,13]. These confounding factors may limit digital grasping, ring forceps extraction, or passage of a Foley beyond the object and can lead to failure of Emergency Department (ED) recovery of the RFB [4,6,13]. Failure of bedside ED recovery of RFBs may result in more invasive extraction methods, including the utilization of rigid sigmoidoscopy and surgical interventions that are associated with higher rates of complications [2,7,14-18]. Therefore, different approaches to removing a low-lying RFB and avoiding more invasive methods will benefit patients [1].

Foley catheters have been used in the removal of RFBs. Many published case reports propose the passing of the Foley balloon beyond the offending object, followed by inflation and traction [8,19]. However, few articles in the literature describe the inflation of a Foley balloon within an object [15,20]. We report a unique and challenging case of a patient who underwent a bedside procedural sedation along with the removal of a large homemade RFB using an intraluminal placement of a Foley catheter technique. This study was approved by the Institutional Review Board (IRB) at Arrowhead Regional Medical Center with the IRB approval number 11-28.

## Case presentation

A 51-year-old male presented to the ED with a chief complaint of abdominal pain and nausea, accompanied by constipation for the past three days. The patient stated that it "might have been because there is a sex toy in my rectum." According to the patient, the RFB was inserted three days before his presentation and could not be retrieved at home. In addition, he has been experiencing increasing diffuse, sharp abdominal pain with cramping, no bowel movement, or flatulence for the past three days.

The patient denied any past medical history, surgeries, medications, or allergies. He admitted to using marijuana occasionally but denied tobacco or alcohol use. On the physical examination, blood pressure was 134/88 mmHg, heart rate 85 beats per minute, respiratory rate 16 breaths per minute, temperature 98.2 degrees Fahrenheit, and oxygen saturation was 99% on room air. He appeared calm, alert, and in mild distress. During the evaluation and a lengthy discussion regarding possible treatment plans, the patient would not reveal the nature of the inserted RFB.

On physical examination, the abdomen was soft with mild tenderness to palpation diffusely. On deep palpation of his abdomen, a firm mass in the left lower quadrant was notable. No gross blood or perianal soft tissue injury was noted on the rectal examination. Furthermore, the rectal vault was noted to be empty. The rest of his physical examination was unremarkable.

**Figure 1:**
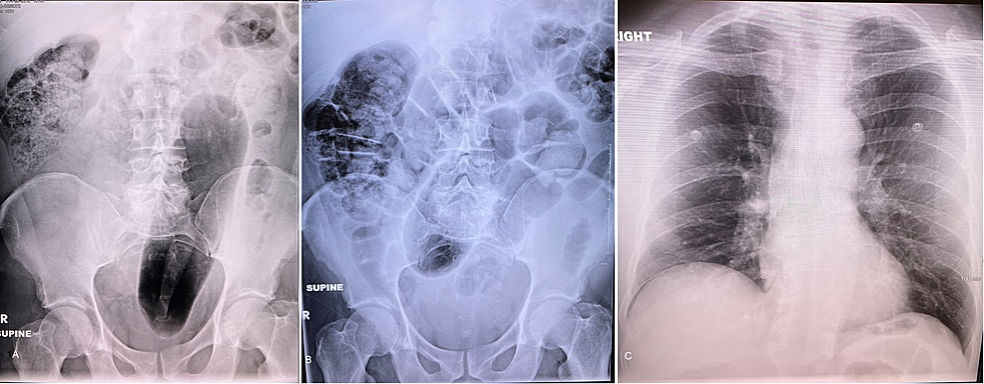
Abdominal and chest X-Ray: A. Abdominal X-ray image with a large area of hyperlucency suspicious for a foreign body in the colon. B. Repeated abdominal X-ray image shortly after the removal of the RFB. C. Chest X-ray image after removing RFB and before discharge. RFB, rectal foreign body.

After the initial evaluation, an abdominal X-ray indicated a suspicious radiopaque foreign object in the descending colon and distal rectal vault (Figure 1A). There was no evidence of free air or obstruction. Upon further discussion regarding various treatment options, the patient consented to undergo procedural sedation and bedside exploration. In addition, because of the complexity of the patient's condition and in anticipation of possible complications leading to possible emergent surgical intervention, all necessary consents were obtained from the patient before the bedside attempt in removing the RFB. Subsequently, the patient was placed on a monitor with supplemental oxygen, end-tidal carbon dioxide monitoring, and a total of 100 milligrams of propofol and 50 milligrams of ketamine was intravenously administered in small increments. At the same time, the emergency medicine and general surgery team attempted rectal exploration.

The patient’s hips were fully flexed toward his chest, and external compression was applied on the superior border of the object palpated over the left upper quadrant of his abdomen. On the further attempt, the inferior border of the RFB was palpable intra-rectally deep in the descending colon. All conventional methods, including digital manipulation, rigid scopes, and inability to pass a urinary catheter around the object, failed to dislodge the foreign body. The object seemed to be set firmly and tightly in the inner parameter of the descending colon, and any forceful manipulation could have damaged the colonic wall. After several unsuccessful attempts, an experienced ED attending physician was able to palpate the RFB on his digital exam and noted it to be hollow and round with smooth lumen.

**Figure 2:**
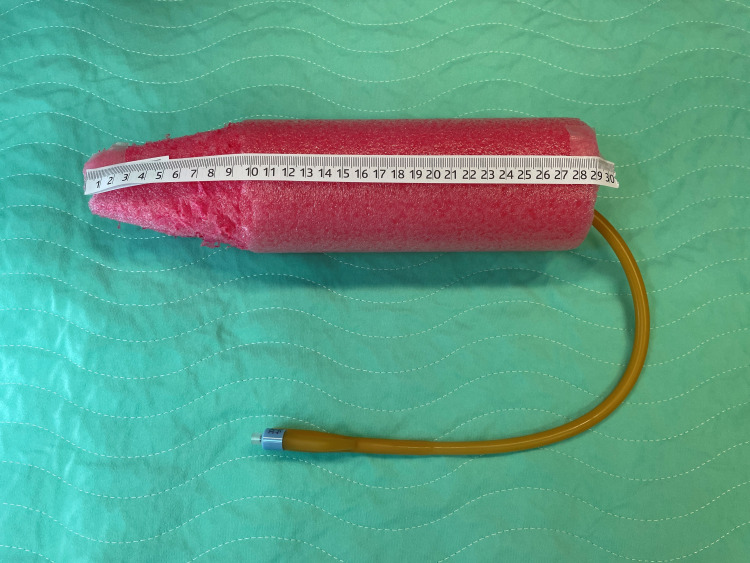
The rectal foreign body with associated Foley catheter and ruler for scale

During the last attempt before transferring the patient to the surgical suite for surgical exploration, a 24 French urinary catheter was inserted through the object's central opening. Subsequently, 20 mL of normal saline was used to inflate the catheter balloon to create a tight seal between the Foley catheter balloon and the inner wall of the object’s central opening. Finally, with a steady, mild downward traction, the object started to move and was extracted slowly through the rectal vault. The homemade sex toy measured 28 cm in length and 3.25 cm in diameter (Figure 2). Following the extraction, rigid sigmoidoscopy equipment was used to immediately assess the state of the patient's rectum and descending colon. In addition, a follow-up kidney, ureter, and bladder (KUB) and chest X-ray confirmed the removal of the RFB, along with no evidence of free air in the abdominal cavity (Figure 1B, 1C). The patient was observed in the ED for 6 h and discharged in good condition to follow up as an outpatient.

## Discussion

To reduce the complications in removing RFBs, differences in the shape, size, and materials of objects should be considered. Many RFBs are challenging to grasp, and various approaches without the use of sigmoidoscopy or endoscopy have been described [15,17,18,20-29]. It has been noted that an object with sufficiently large size or glass-made products, in particular, can put the patient at a higher risk of bowel perforation [30-32]. As a result, physicians should approach each case of RFB carefully and consider the shape, location, and composition of the object prior to an attempt to remove it. In most cases, RFB can be retrieved using various transanal techniques [9].

Foley catheters have been used in the removal of the RFBs. Table 1 summarizes some of the reported utilization of Foley catheters in the removal of the RFB encountered in the literature. In most reported techniques, the catheter is passed by the object and placed beyond the RFB rather than within the object [8,19,33,34]. In addition, a few published reports have described removing glass-made RFBs by inflating Foley catheters within the parameter of the objects. However, those occurred under general anesthesia and in the surgical setting in which the use of deep sedation and paralytics may help with relaxation of colonic structure [15,20]. Lee and his colleagues also suggested that the use of propofol may help relax the colonic smooth muscle wall during the extraction procedures [35].

**Table 1:** Case reports utilizing rectal Foley catheters to facilitate extraction of RFBs. *Inflating the Foley catheter specifically within the RFB.  ^†^Inflating the Foley catheter outside and past the RFB. ^‡^Using the Foley catheter to inject air behind the RFB. RFBs, rectal foreign bodies.

Type foreign body	Technique	Anesthesia	Disposition outcome	Complications	Author
Perfume bottle	Size 24 Foley catheter^†^	General	Discharged after period of observation	No complications	Bakheit et al. [8]
Foreign body	Foley catheter number 20^†^	General	Discharged after period of observation	No complications	Shiryazdi and and Jabbari [19]
Mandarin orange	Foley catheter 14 French^†^	General	Discharged after period of observation	No complications	Sayılır et al. [33]
Rubber ball diameter 6 cm	#18 Foley catheter^‡^	General	Discharged after period of observation	No complications	Nivatvongs et al. [34]
Bottle neck	Inflated 14 French Foley catheter*	General	Discharged after period of observation	No complications	Humes and Lobo [20]
Glass jar	Inflated Foley catheter*	General	Discharged after period of observation	No complications	Yaman et al. [15]

The wide variety of RFBs and their effects on local colorectal tissue require a systematic approach to devise an extraction strategy [24,36,37]. A detailed physical and digital examination combined with abdominal imaging studies such as X-ray and computed tomography (CT) scan can help evaluate and locate the foreign body resulting in a more reasonable approach for each specific patient's condition [38]. If the physical examination or imaging indicates evidence of peritonitis or perforation, any manipulation of the RFB should be avoided and surgical intervention should be immediately considered [37]. On the other hand, before resorting to endoscopy or surgery, a detailed digital examination may determine whether an attempt to grasp the object manually is possible. Among the arsenal of tools for the bedside removal of the RFB, a Foley catheter may be considered by passing beyond the RFB, the Foley balloon subsequently inflated and slowly and steadily pulled back out of the rectal vault opening along with the object [39].

Potential complications from the use of a Foley may include pressure necrosis resulting from compression against the visceral wall from increased pressure of the inflated balloon, which may ultimately cause perforation [40,41]. The anal canal mucosa is composed of columnar epithelial cells, which thinly overlie the hemorrhoidal venous plexuses, contributing to a massive hemorrhagic risk [42]. The structural composition of lower gastrointestinal mucosa warrants additional caution when considering the removal of RFBs.

Utilizing the Foley catheter technique by placing the catheter balloon within the inner space of the hollow with a continuous channel through the objects may decrease the likelihood of inflicting abnormal pressure directly to the visceral wall, therefore avoiding pressure necrosis. However, in some cases of RFB made of soft-shell, the pressure necrosis could still be inflicted if the balloon expands the outer wall of the soft shell RFB, transferring the pressure into the adjacent visceral walls.

## Conclusions

RFBs may pose a unique challenge. A detailed understanding of the composition and structure of the RFB itself can help devise the best plan for extraction and avoid further complications. Placing the Foley catheter balloon within the inner space of the RFB can successfully remove the RFB without further complications. Various uses of Foley catheters may provide safe and alternative modes of extraction of hollow objects.
